# Effect of pilocarpine on the formalin-induced orofacial pain in rats

**Published:** 2012

**Authors:** Esmaeal Tamaddonfard, Amir Erfanparast, Emad Khalilzadeh

**Affiliations:** *Department of Basic Sciences, Faculty of Veterinary Medicine, Urmia University, Urmia, Iran.*

**Keywords:** Pilocarpine, Atropine, Naloxone, Orofacial pain, Rat

## Abstract

In this study, the effects of subcutaneous (SC) injection of pilocarpine (a cholinomimetic agent) and atropine (a muscarinic receptors antagonist) were investigated on a tonic model of orofacial pain in rats. The contribution of the endogenous analgesic opioid system was assessed using naloxone (an opioid receptors antagonist). Tonic orofacial pain was induced by SC injection of a diluted formalin solution (1%, 50 μL) in the right upper lip, and the time spent face rubbing was measured in five min blocks for 1 h. Formalin induced a biphasic (first phase: 0-5 min and second phase: 15-35 min) pain response. Pilocarpine significantly (*P *< 0.05) suppressed both phases of orofacial pain. Atropine did not have any effect and naloxone non-significantly increased the intensity of pain when used alone. In the pre-injection examinations, atropine prevented, but naloxone did not reverse the antinociceptive effect of pilocarpine. The results indicated that SC injection of formalin in the orofacial region induced a marked biphasic pain. Pilocarpine via muscarinic cholinergic receptors produced antinociceptive effect in the orofacial formalin-induced pain. The endogenous opioid analgesic system may not have a role in pilocarpine-induced antinociception.

## Introduction

The orofacial region is one of the most densely innervated (e.g. the trigeminal nerve) areas of the body, which focuses some of the most common acute pain, i.e. those accompanying the pathological states of the teeth and the related structures. It is also the site of frequent chronic (post-herpetic neuralgia and migraine) and referred pain.^1^ The orofacial formalin test was introduced as a tonic model of pain,^2^ and thereafter has been frequently used with success in the study of modulating mechanisms of orofacial pain.^3^^-^^5^

The role of acetylcholine, cholinergic agonists and cholinesterase inhibitors, collectively termed cholinomimetics, in the modulation of pain and analgesia has been established.^6^ Although there is no report showing the effect of systemic injection of pilocarpine on pain, the involvement of other cholinomimetics such as physostigmine, neostigmine on pain and analgesia was reported.^7^^,^^8^ Moreover, the antinociceptive effects induced by intra-hippocampal and intra-dentate gyrus administration of pilocarpine were reported.^9^^,^^10^

Naloxone is a competitive antagonist of mu and kappa receptors with higher affinity for mu receptors.^11^ Naloxone has been used to explore the involvement of endogenous opioid system in the rat model of orofacial region pain.^12^^-^^14^

The aim of the present study was to investigate the effects of pilocarpine and atropine on the orofacial pain induced by subcutaneous injection of formalin in the upper lip in rats. Moreover, the involvement of the endogenous analgesic opioid system on the effect of pilocarpine in pain was assessed with subcutaneous (SC) injection of naloxone.

## Materials and Methods

Healthy adult male Wistar rats, weighing 230-270 g were used in this study. Rats were maintained in polyethylene cages with food and water available *ad libitum*, with controlled ambient temperature (23 ± 0.5 °C) and under a 12 h light-dark cycle (lights on at 07:00 h). Six rats were used in each drug treatment. Experiments were carried out between 12:00 h and 16:00 h. The experimental protocol was approved by the Laboratory Animal Care and Use Center of the Faculty of Veterinary Medicine of Urmia University.

The drugs used in the present study were pilocarpine, atropine sulfate and naloxone dihydrochloride. All drugs were purchased from Sigma-Aldrich Co., Steinheim, Germany, and were dissolved in normal saline. Pilocarpine at doses of 0.125, 0.25, 0.5, 1, 2, and 4 mg kg^-1^, atropine (1 mg kg^-1^) and naloxone (1 mg kg^-1^) were SC injected using a 27-gauge injection needle at the back region of the neck. Pilocarpine, atropine and naloxone were injected 30, 20, and 15 min before induction of orofacial pain, respectively.

The doses of pilocarpine, atropine and naloxone used in the present study were close to other reports.^15^^-^^18^

Orofacial formalin test was used for the induction of pain. Before the rats were pain-tested, they were placed in plexiglass chambers (30 × 30 × 30 cm) for 30 min on three successive days to minimize stress-activated pain suppressive mechanisms.^19^ The orofacial formalin test was applied as follows. Fifty microliters of 1% diluted formalin solution was SC injected in the right upper lip just lateral to the nose using a 30-gauge injection needle.^5^ The rat was placed in a chamber with a mirror mounted at 45 degrees angle beneath the floor to allow an unobstructed view of the orofacial region. 

The time each animal spent facial rubbing with ipsilateral forepaw was recorded (using a stopwatch), in consecutive 5-min bins over a period of 1 h, and was considered as an index of nociception. Formalin injection induced a stereotyped response characterized by two well distinct phases.^1^^,^^5^^,^^20^ In the present study, data collected between 0 and 5 min post-formalin injection represented first (early) phase and data collected between 15 and 35 min after injection of formalin represented second (late) phase. 

Data obtained from the SC injections of normal saline and formalin were analyzed using repeated measure ANOVA followed by Duncan’s test. To evaluate significance differences among drug-treated groups, one-way analysis of variance (ANOVA) and Duncanۥ s test were applied. In figures, all values are expressed as the mean ± SEM. A value of *P *< 0.05 was considered statistically significant.

## Results

**Fig. 1:**
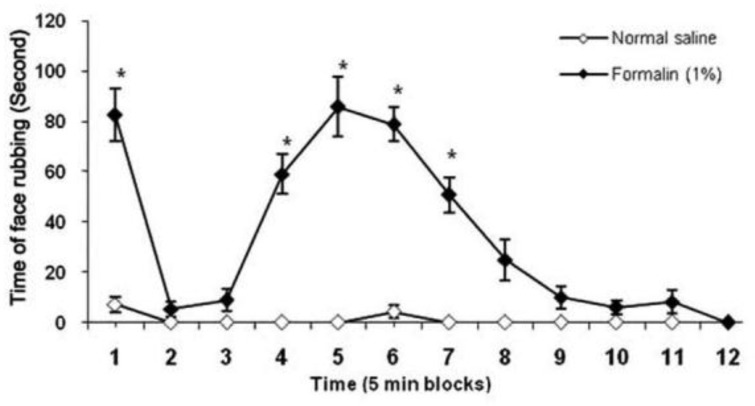
The orofacial pain response induced by SC injection of normal saline and formalin in the upper lip in rats. Each point represents the mean ± SEM. (n = 6). * indicates significant difference compared with normal saline and other 5-min blocks (*P *< 0.05).

The SC injection of normal saline into the rat upper lip produced a negligible nociceptive response only in the first 5-min block (Fig. 1). Diluted formalin, when injected SC into the upper lip, produced a typical pattern of face rubbing behavior. Significant differences in face rubbing were observed among 1^st^, 4^th^, 5^th^, 6^th^, and 7^th^ with the other 5-min blocks after subcutaneous injection of formalin (*P *< 0.05).

Therefore, the formalin-induced nociceptive behavior showed a biphasic time course: the first phase began immediately after formalin injection and declined in approximately 5 min, while the second phase began about 15 min after formalin injection and lasted about 20 min and declined to the end of recording period (1 h) (Fig. 1). 

**Fig. 2:**
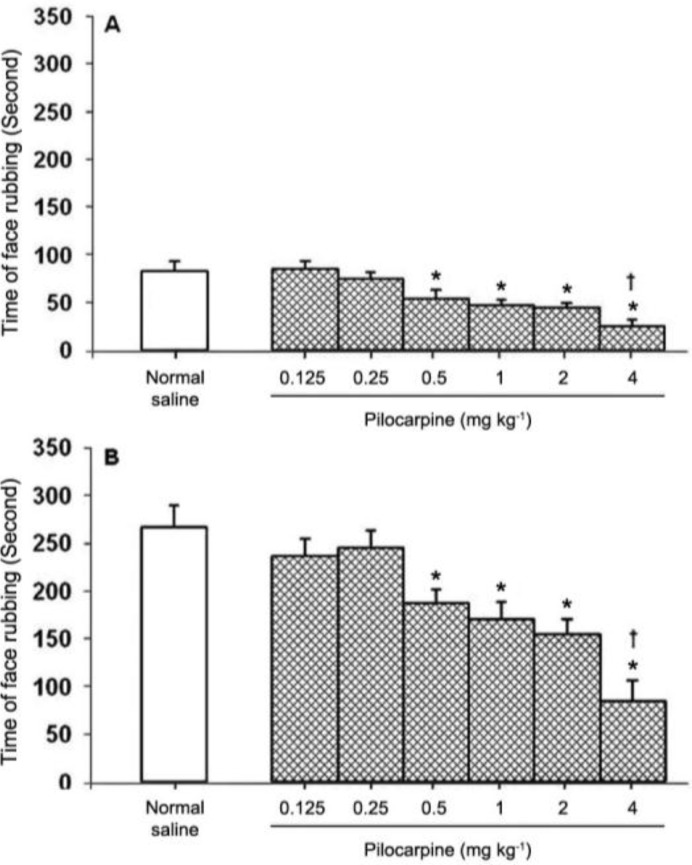
Effect of pilocarpine on the first (A) and second (B) phases of formalin-induced orofacial pain. Each column represents the mean ± SEM. (n = 6). * indicates significant difference compared with normal saline treated group (*P *< 0.05), ^†^ indicates significant difference compared with pilocarpine (0.125, 0.25, 0.5, 1, and 2 mg kg^-1^) treated groups

The SC injection of pilocarpine at doses of 0.125, 0.25 mg kg^-1^ had no effect, whereas at doses of 0.5, 1, 2, and 4 mg kg^-1^ pilocarpine significantly (*P *< 0.05) suppressed both phases of pain. No significant differences were observed among the antinociceptive effects induced by 0.5, 1, and 2 mg kg^-1^ of pilocarpine. The antinociceptive effect induced by SC injection of 4 mg kg^-1^ of pilocarpine was more than that obtained from pilocarpine at doses of 0.5, 1, and 2 mg kg^-1^ (Fig. 2A and 2B). 

**Fig. 3:**
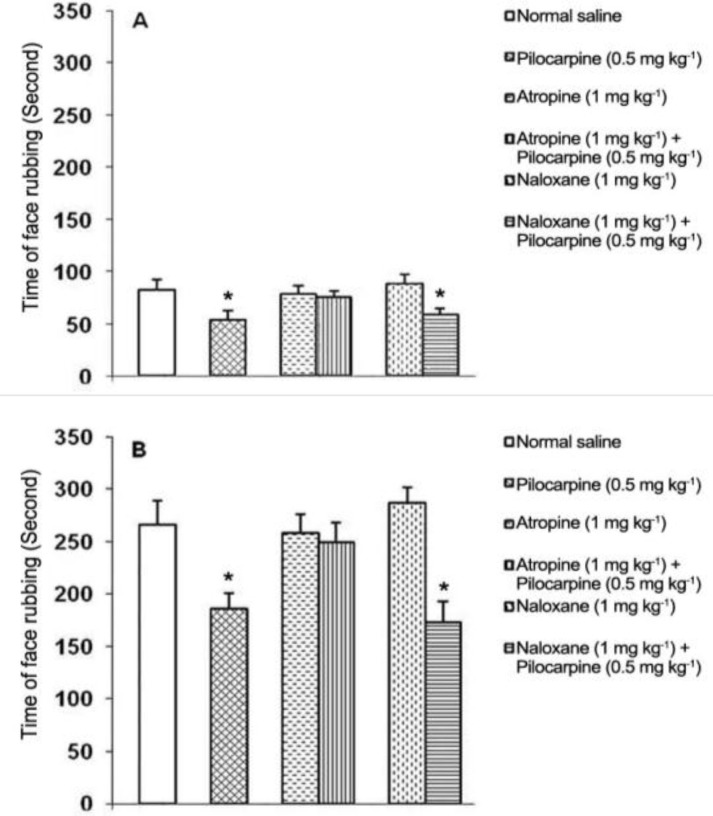
Effects of atropine and naloxone on the pilocarpine-induced antinociception in the first (A) and second (B) phases of orofacial formalin test. Each column represents the mean ± SEM. (n = 6). * indicates significant difference compared with other treated groups (*P* < 0.05).

The SC injections of atropine and naloxone at the same dose of 1 mg kg^-1^ alone had no effect, whereas pretreatment with atropine (1 mg kg^-1^) significantly (*P *< 0.05) blocked the pilocarpine-induced antinociceptive effects on the first and second phases of pain. The SC injection of naloxone (1 mg kg^-1^) after pilocarpine (0.5 mg kg^-1^) did not reverse pilocarpine-induced antinociception on both phases of pain (Fig. 3A and 3B). 

## Discussion

The present study shows that the SC injection of formalin into the upper lip produced a distinct biphasic pattern in the face rubbing performed by ipsilateral forepaw. The SC injection of formalin 1.5% into the upper lip induced a biphasic pattern in the face rubbing in rats.^21^ During the orofacial formalin test, two distinct phases due to different mechanisms of nociception produces the first phase which is associated with direct stimulation of C-nociceptors, and the second phase which is a reflection of integration between nociceptors and spinal and brainstem signaling.^22^ Face rubbing with the ipsilateral forepaw due to formalin injection into the upper lip has been mentioned as a specific nociceptive response.^1^ Vocalization, grooming and scratching due to electrical, mechanical, thermal and chemical (capsaicin, formalin) stimulation of the orofacial region have been reported by some researchers.^23^^-^^26^ However, nociceptive behavior obtained from the present study is in agreement with other investigations.^1^^,^^5^^,^^21^^,^^27^^,^^28^

The effect of pilocarpine on the orofacial formalin-induced pain, at least in the present study, was not dose dependent. Pilocarpine at low doses (0.125 and 0.25 mg kg^-1^) had no effect. There was no significant difference observed between the antinociceptive effects induced by pilocarpine at the doses of 0.5, 1, and 2 mg kg^-1^. The antinociceptive effect produced by pilocarpine at a dose of 4 mg kg^-1^ was at the highest level. Pilocarpine, as a cholinomimetic agent at the dose range of 1-8 mg kg^-1^, has been frequently used in the study of the involvement of the cholinergic system in behavioral and physiological events such as jaw tremor, yawning and salivation in rats.^15^^,^^16^ Therefore, the high antinociceptive response induced by pilocarpine at the high dose observed in the present study might have been associated with induction of other events such as tremor interfering with pain mechanisms.

In other words, pilocarpine-induced other behaviors such as salivation, tremor, yawning, and defecation may be interrupted the pain behavior such as face rubbing. The preventive effect of atropine on the pilocarpine-induced antinociception produced in the present study indicated that muscarinic receptors might be involved in the antinociceptive effect of pilocarpine. There is no report describing the antinociceptive effect induced by systemic injection of pilocarpine in the formalin test in rats. However, intra-hipocampal and intra-dentate gyrus microinjection of pilocarpine produced antinociceptive effect in anesthetized rats. The antinociceptive effect induced by pilocarpine was inhibited by pretreatment with atropine.^9^^,^^10^

In the present study, naloxone non-significantly increased the pain intensity, and pilocarpine-induced antinociception was not reversed with naloxone. This indicates that pilocarpine-induced analgesia was not mediated through endogenous analgesic opioid system. There is not any report describing the mechanism of pilocarpine-induced antinociception. The analgesic effect induced by SC injection of physostigmine, a cholinomimetic agent, was not antagonized with naloxone in the neuropathic pain in rats.^29^ However, intrathecal injection of physostigmine and neostigmine with morphine produced synergistic antinociceptive effects in the hot plate and tail immersion tests of nociception in rats.^30^ In the present study, we used the 0.5 mg kg^-1^ of pilocarpine in the pretreatment examinations. This may be associated with the fact that statistical analysis did not show any significant differences among the anti-nociceptive effects produced by 0.5, 1, and 2 mg kg^-1^ of pilocarpine. On the other hand, the beginning of pilocarpine-induced behaviors such as tremor, yawning and salivation was observed when 1 and 2 mg kg^-1^ of pilocarpine was used. In this study, we observed, but not recorded, pilocarpine-induced behaviors including tremor, salivation, defecation, and yawning.

In conclusion, results of the present study showed that pilocarpine, through muscarinic cholinergic receptors produced an antinociceptive effect in formalin-induced oro-facial pain. The endogenous analgesic opioid system might not be involved in pilocarpine-induced antinociception.
